# Potential Biological Targets of Anticancer Metal‐Based Drug Candidates: A Systematic Review

**DOI:** 10.1002/ddr.70315

**Published:** 2026-05-18

**Authors:** Allysson L. dos S. Ferreira, Bruna B. Dantas, Jailton De Souza‐Ferrari, Edilson B. Alencar Filho

**Affiliations:** ^1^ Graduate Program in Biosciences Federal University of Vale do São Francisco Petrolina Pernambuco Brazil; ^2^ Academic Health Unit/Center for Education and Health Federal University of Campina Grande Cuité Paraíba Brazil; ^3^ Department of Chemistry, Center for Exact and Natural Sciences Federal University of Paraíba, Campus I Castelo Branco Paraíba Brazil; ^4^ Pharmaceutical Sciences Program Federal University of Vale do São Francisco Petrolina Pernambuco Brazil; ^5^ Graduate Program in Health and Biological Sciences Federal University of Vale do São Francisco Petrolina Pernambuco Brazil

**Keywords:** anticancer activity, biological targets, metal complexes

## Abstract

The discovery of Cisplatin marked the beginning of the metallodrug era in oncology. Despite their clinical success, platinum‐based compounds present important limitations, including drug resistance and systemic toxicity. These challenges have stimulated interest in alternative metal complexes capable of interacting with biomolecular targets beyond DNA, particularly proteins involved in cancer‐related pathways. To systematically identify and categorize protein targets associated with the antitumor activity of metal complexes in preclinical studies, emphasizing their functional classification and biological relevance. A systematic search was conducted in Web of Science, Wiley, Scopus, and ScienceDirect for studies published between January 2015 and March 2025. Original studies evaluating the antitumor activity of metal complexes with experimental or computational evidence of protein‐directed mechanisms were included. Reviews, editorials, theoretical studies without experimental support, and studies focused exclusively on serum albumin binding were excluded. Of the 873 records identified, 59 studies met the inclusion criteria. Reported targets were organized according to Gene Ontology–based biological processes, revealing recurrent associations with redox regulation, apoptosis and cell‐cycle control, and DNA replication and repair, with fewer studies addressing angiogenesis and drug‐resistance mechanisms. Across categories, mechanistic evidence is predominantly derived from in vitro assays and computational analyses, with limited demonstration of selective intracellular target engagement. Current evidence indicates that metal complexes frequently perturb survival‐related cellular networks, particularly those associated with redox balance and stress‐response pathways. However, the available literature remains largely preclinical and mechanistically heterogeneous, highlighting the need for more rigorous target‐validation strategies to clarify the therapeutic relevance of proposed protein targets.

## 1 Introduction

The discovery of cisplatin by Rosenberg in 1965 (Muggia et al. 2015) marked the beginning of the metallodrug era in oncology, laying the foundation for developing new antitumor compounds (Kumar Singh et al. 2023). Although cisplatin and its derivatives (oxaliplatin, carboplatin) remain mainstays in treating several cancers (Romani 2022), their efficacy is limited by tumor resistance, mediated by mechanisms such as altered cellular transport, increased detoxification, enhanced DNA repair, and changes in the tumor microenvironment (Fu et al. 2023). Their low selectivity also causes significant adverse effects, including nephrotoxicity and myelosuppression (Oun et al. 2018).

These limitations have spurred interest in alternative metal complexes involving ruthenium, gold, palladium, and copper (Benjamin garbutcheon‐Singh et al. 2011; Abdolmaleki et al. 2024; León 2024). Owing to their redox properties, molecular geometries, and coordination versatility, these metals have been associated with a range of reported mechanisms, including interactions with DNA, inhibition of specific proteins and enzymes, induction of reactive oxygen species (ROS), and perturbation of cellular signaling pathways (Karges et al. 2021; Abdolmaleki et al. 2024).

Platinum chemotherapeutics act mainly by forming DNA adducts, a mechanism that, while effective, causes genomic damage and toxicity in healthy tissues (Van den Boogaard et al. 2022). In contrast, metal complexes targeting specific proteins may overcome low selectivity and side effects. Many also tend to accumulate in tumors, potentially enhancing treatment efficacy (Peng et al. 2023). Consequently, there is a growing scientific interest in metal complexes that act on targets other than DNA, offering a promising frontier for safer, more effective antineoplastic agents, which offer a promising frontier for safer (Steel and Hartinger 2020).

This systematic review aims to catalog the molecular targets of antitumor metal complexes, analyze trends, and identify research gaps to support the rational development of new, safer metal‐based therapies.

## 2 Methods

This systematic review was conducted according to the PRISMA 2020 guidelines (Page et al. 2021). The guiding question—“Which proteins associated with tumor progression have been identified as molecular targets of metal complexes with antitumor activity in preclinical studies?”—guided the search and evaluation of selected articles. The PRISMA checklist was completed and will be made available as Supporting Information.

### 2.1 Eligibility Criteria

Original studies published between January 2015 and March 2025, without language restrictions, that proposed and evaluated interactions between metal complexes and proteins associated with tumor progression using in vitro and/or in vivo experimental models were included. Computational studies were considered when the theoretical results supported experimental findings. The following were excluded:

- Exclusively theoretical studies lacking adequate validation or rationale;
- Studies without discussion of mechanisms of action involving target proteins;
- Reviews, editorials, commentaries, and other nonoriginal publications;
- Studies without full‐text availability;
- Studies focused solely on interactions with bovine serum albumin (BSA).

The primary aim of this review was to qualitatively map the landscape of protein targets rather than perform a quantitative meta‐analysis of compound potency. The marked heterogeneity of the available literature—including diverse metal complexes, cell models, and experimental protocols—precludes meaningful statistical pooling or direct comparison of quantitative parameters such as IC₅₀ values or docking energies. Accordingly, this review emphasizes a systematic and critical synthesis of the reported molecular targets.

Although bovine serum albumin (BSA) is widely used as a model protein in metallodrug research due to its relevance to pharmacokinetics and systemic distribution (Topală et al. 2014), its primary biological function is related to compound transport rather than direct involvement in tumorigenic pathways. Interactions between metal complexes and albumin have been extensively investigated (Bashir et al. 2022; Pandya and Sivaramakrishna 2024; van Niekerk et al. 2024), particularly in relation to bioavailability and circulation time. Because this review focuses specifically on proteins implicated in tumor biology and mechanistic antitumor activity, studies exclusively examining BSA binding were excluded. Nevertheless, albumin binding and metal complex speciation remain important pharmacological factors that may influence intracellular target accessibility and therapeutic outcomes.

### 2.2 Sources of Information

Searches were performed in the Web of Science, Wiley, Scopus and ScienceDirect databases, covering publications from January 2015 to March 2025. The last search was conducted in March 2025.

### 2.3 Search Strategy

Standardized descriptors from MeSH and DeCS vocabularies (“Coordination Complexes,” “Antineoplastic Agents”) were combined with free keywords such as “anticancer activity,” “metallodrugs,” “molecular dynamics,” “mechanism of action,” “binding affinity,” and “docking,” using the Boolean operators “AND,” “OR,” and “NOT.” Search expressions were adapted for each database according to their indexing systems. Searches in PubMed/Medline and Embase retrieved a limited number of eligible studies under the predefined criteria, likely reflecting differences in database indexing for chemically oriented metallodrug research. The complete strategy is detailed in the Supporting Information (Table S1).

### 2.4 Study Selection Process

Study selection was conducted by a single reviewer using the Rayyan platform in accordance with PRISMA guidelines. We acknowledge that the single‐reviewer approach represents a limitation, as it precludes assessment of inter‐rater agreement and may introduce selection bias. To mitigate this limitation, the screening protocol was designed to maximize objectivity and consistency. Eligibility criteria were defined using binary and minimally subjective conditions (e.g., mandatory inclusion of direct protein target evidence and explicit exclusion criteria).

Screening followed an iterative two‐stage procedure. After the initial screening, all excluded records were re‐evaluated by the same reviewer after a predefined interval to identify potential oversights and confirm adherence to the eligibility criteria. This self‐verification step served as an internal quality control measure within the single‐reviewer framework. The screening log is available upon request. Finally, the manuscript and the rationale for inclusion decisions were reviewed and approved by all co‐authors, providing additional oversight of the review scope and conclusions.

### 2.5 Data Collection Process

A single reviewer extracted data after full‐text reading, using a structured spreadsheet with two main categories: (1) primary data (Table S2), including molecular targets, metal centers, mechanisms of action, and full references; and (2) an analytical synthesis (Table 2), grouping targets by biological function or cellular process, along with associated metals and study counts. This classification aimed to highlight patterns and visualize trends. The entire process was systematically documented to ensure traceability.

### 2.6 Data Items—Main Outcomes

The following were extracted:

- Specific molecular targets (e.g., enzymes, DNA) and their functional classification (redox, apoptotic pathways, etc.);
- Proposed mechanisms of action (e.g., Increased ROS, DNA damage);
- Frequency of identification of each target in the included studies.

### 2.7 Data Items—Other Variables

The following were collected:

- Metal centers (e.g., Pt(II), Ru(III));
- Experimental/computational techniques (e.g., molecular docking, enzymatic assays);
- Type of validation (biological, in silico, or both).

### 2.8 Methodological Quality Assessment

The methodological quality of the included studies was assessed using a tailored checklist, as no standardized risk‐of‐bias tool exists for the highly heterogeneous preclinical data characteristic of this field. Following the logic proposed by Sargeant et al. (2022), the assessment focused on three fundamental criteria that indicate the completeness of the evidentiary framework supporting a proposed protein target:

- Direct Experimental Evidence: Provision of experimental data (e.g., enzymatic assays, biophysical techniques) reported as demonstrating target engagement.
- Supporting Computational Evidence: Use of in silico methods to propose or characterize protein–ligand interactions.
- Correlation with Biological Activity: Demonstration of a functional biological effect plausibly associated with the proposed target.

Each study was classified with a binary “Yes” or “No” for each criterion. The full study‐level assessment is provided in Supplementary Table S3, and a summary of the aggregated results is presented in Table 1. In this review, “methodological quality” is operationally defined as the completeness of complementary evidence types, rather than a formal appraisal of technical rigor or target specificity. This approach categorizes the type and breadth of reported evidence but does not evaluate internal validity or susceptibility to metal‐specific artifacts, a distinction critical for the subsequent synthesis and discussion.

**Table 1:** Summary of methodological quality assessment across the 59 included studies.

Methodological Quality Criterion	No. of Studies Fulfilling Criterion (n/N)	Percentage
Direct Experimental Evidence	42/59	71%
Supporting Computational Evidence	40/59	68%
Correlation with Biological Activity	52/59	88%

## 3 Results and Discussion

### 3.1 Study Selection

**Figure 1:**
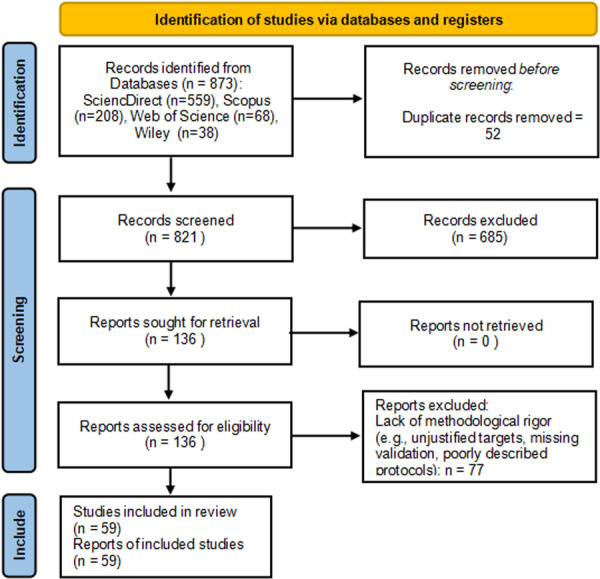
Flowchart of the process of identification, screening, eligibility and inclusion of studies, according to the PRISMA 2020 guidelines. *Source:* By the author, 2026.

The database search identified 873 articles: 559 from ScienceDirect, 208 from Scopus, 68 from Web of Science, and 38 from Wiley. After removing 52 duplicates in Rayyan, 821 unique records remained. Title and abstract screening yielded 136 articles meeting the inclusion criteria. After full‐text evaluation, 77 were excluded for being exclusively theoretical without experimental validation or justification. Ultimately, 59 studies were included for discussion, as shown in the flowchart in Figure 1.

### 3.2 Characteristics of Included Studies

The 59 studies included in this review examine interactions between metal complexes and protein targets implicated in cancer. The methodological approaches are heterogeneous, ranging from direct biochemical assays (e.g., enzyme inhibition and spectroscopy) to computational modeling and indirect cellular readouts, with only a small number of studies relying exclusively on theoretical analyses.

To synthesize this diverse evidence and enable transparent comparison across targets, the studies were grouped by molecular target (Table S2), which summarizes the evidence base underlying the pathway‐oriented analysis presented below. For each target, the table includes a “Level of Evidence” classification derived from two factors: (1) the number of independent studies reporting the interaction and (2) the robustness of the methodological approaches used for target identification, as assessed by the tailored quality checklist described in the Methods.

This classification reflects recurrence and methodological convergence within the literature rather than definitive biological validation. A “Strong” classification indicates repeated reporting supported by direct experimental assays within the included studies but does not imply low risk of bias, selective intracellular engagement, or causal relevance to antitumor activity. This distinction is particularly important in metallodrug research, where in vitro enzymatic assays may be susceptible to metal‐induced interference, redox artifacts, or nonspecific protein reactivity.

Accordingly, targets classified as “Strong” (e.g., thioredoxin reductase) should be interpreted as consistently reported interaction sites rather than unequivocally established primary drivers of cytotoxicity. Conversely, a “Preliminary” classification refers to interactions reported in a single included study and reflects the limited evidence available within this dataset. In such cases, the functional consequences of the reported interaction and its contribution to the observed antitumor effects often remain to be clarified.

### 3.3 Methodological Quality Assessment

The aggregated results of the methodological quality assessment are summarized in Table 1, with the complete, study‐level data available in Table S3.

This quantitative summary highlights predominant methodological patterns within the field. A high proportion of studies (88%) reported a correlation with relevant biological activity, while a majority also employed direct experimental assays (71%) and/or computational approaches (68%). These results describe the composition of the evidence base supporting proposed protein targets rather than the technical rigor of individual studies. Within the scope of this review, the checklist assesses whether a study presents a coherent argument for a proposed target by integrating experimental data, computational modeling, and functional biological outcomes. In this operational context, “quality” refers to the presence of complementary types of evidence.

This metric, however, is intentionally limited. It does not evaluate assay robustness, adequacy of controls, reagent purity, or validation against metal‐specific artifacts. In metallodrug research, this limitation is particularly relevant because many commonly used biochemical and biophysical assays are susceptible to nonspecific metal interactions and assay interference (Groessl and J. Dyson 2011; Kellett et al. 2019; Gerstberger et al. 2024). Consequently, the presence of a “direct” assay should not be interpreted as confirmation of target specificity or high confidence in the proposed mechanism. Even formally direct assays may overestimate target engagement when methodological limitations are not fully addressed.

These considerations affect how the compiled targets should be interpreted. Although the results indicate that the field relies predominantly on empirical validation approaches, many studies test predefined hypotheses regarding specific proteins. Such targeted strategies may overlook off‐target interactions or broader protein engagement profiles that arise from the intrinsic reactivity of metal complexes.

To strengthen confidence in target identification, future studies will likely benefit from the increasing adoption of untargeted discovery strategies. Chemical proteomics approaches, including activity‐based protein profiling (ABPP) and affinity‐based pull‐down assays, provide systematic frameworks for target deconvolution (Meissner et al. 2022; Gao et al. 2023). These techniques enable the unbiased capture and identification of interacting proteins directly from complex biological systems, offering stronger evidence of target engagement in biologically relevant contexts.

Although none of the studies included in this review employed such strategies, their integration represents an important future direction for the field. Expanding beyond a landscape dominated by targeted validation toward one that incorporates untargeted discovery approaches will be essential for validating the targets cataloged here, uncovering additional mechanisms of action, and guiding the development of more selective anticancer metallodrugs.

### 3.4 General Interpretation of the Results

Given the diversity of molecular targets identified across the included studies, proteins were grouped according to shared biological processes using the Gene Ontology (GO) framework (The Gene Ontology Consortium 2018). GO‐based classification was employed as an organizational and interpretative tool to identify recurring biological themes across heterogeneous datasets, rather than to infer pathway‐level causality or validate specific targets as primary drivers of cytotoxicity.

This pathway‐oriented synthesis enables a higher‐level interpretation by shifting the focus from isolated protein–ligand interactions to broader cellular processes most frequently reported as perturbed by antitumor metal complexes. Importantly, this approach does not assume that all cataloged proteins represent selective or primary targets. In the context of metallodrug research, many reported interactions likely reflect downstream consequences of generalized cellular stress—particularly redox imbalance and DNA damage—rather than specific engagement of tumor‐dependent vulnerabilities.

Across the reviewed literature, the most frequently affected biological processes include redox homeostasis, regulation of apoptosis and cell‐cycle progression, and DNA replication and repair (Table 2). These pathways are central to cancer biology and are inherently sensitive to chemically reactive compounds, which may partially explain their predominance. The repeated identification of redox‐related enzymes and stress‐responsive signaling proteins therefore highlights recurrent biological outcomes rather than definitive mechanistic specificity.

**Table 2:** Categorization of protein targets by gene ontology biological processes modulated by antitumor metal complexes.

Biological Process	Related Targets	Metal Center (s)	No. of Studies
Response to oxidative stress (GO: 0006979)	TrxR, GR, Catalase	Au, Ag, Cu, Ru, Pd, Pt, Zn	16
Regulation of apoptotic process (GO: 0042981)/Regulation of cell cycle (GO: 0051726)/Regulation of cell population proliferation (GO:0042127)	BCL‐2 Family, CDKs, p53, LOX, EGFR, HER2, ERα, 20S Proteasome	Ni, Cu, Ag, Mn, Co, Zn, Ru, Pt, Zn, Pd, Cu, Sb, Au	19
DNA damage response (GO:0006974)/DNA replication (GO:0006260)	Topoisomerase I/II, RNR, PARP	Pd, Pt, Cu, Ag, Zn, V, Ru, Ir	9
Negative regulation of angiogenesis (GO:0016525)/Negative regulation of cell migration (GO:0030336)	VEGFR2, Angiogenin, Girdin	Zn, Cd, Pt, Ir	3
Negative regulation of cellular response to drug (GO:2001039)	P‐gp, GSTπ	Ru	2

*Note:* Targets were classified according to the primary antitumor mechanism evidenced in the reviewed studies. In some instances, closely related biological processes (e.g., DNA damage response/DNA replication; negative regulation of angiogenesis/negative regulation of cell migration) are presented together in a single table entry to accurately reflect the interconnected nature of the pathways modulated by the metal complexes, avoiding artificial separation. It is acknowledged that several targets play roles in multiple biological processes; this classification prioritizes the most consistently reported mechanism of action.

**Figure 2:**
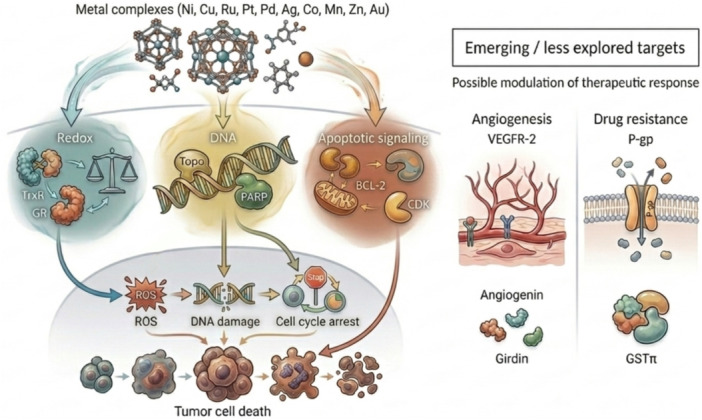
Major biological processes targeted by anticancer metal complexes and their convergence toward tumor cell death. Figure 2. Conceptual overview of the principal biological processes modulated by antitumor metal complexes identified in the reviewed studies. Frequently reported targets converge on three major pathways—redox homeostasis, DNA damage/repair, and apoptosis/cell‐cycle regulation—ultimately contributing to tumor cell death. Emerging targets associated with angiogenesis and drug resistance are shown separately, reflecting their preliminary and less extensively validated status in the current literature. *Source:* By the author, 2026.

Targets associated with angiogenesis and drug resistance were less frequently reported but were retained to provide a comprehensive overview of emerging research directions. In most cases, evidence supporting these targets remains preliminary, and their inclusion should be interpreted as indicative of exploratory interest rather than validated therapeutic relevance. A conceptual overview of how the principal biological processes identified in Table 2 converge toward tumor cell death is presented in Figure 2.

Collectively, this synthesis reveals a field characterized by strong chemical innovation but limited biological resolution. The distinctive coordination chemistry of metal complexes enables interactions with biologically challenging targets but also introduces important pharmacological constraints that influence selectivity, target engagement, and translational interpretation. These limitations are discussed in greater detail in the section “Pharmacological and target‐selectivity considerations.” Accordingly, the GO‐based framework presented here should be understood as a descriptive map of recurrent biological perturbations, rather than confirmation of selective or clinically actionable target engagement.

Beyond chemical reactivity and target classification, an important biological dimension concerns whether the frequently reported protein targets correspond to genuine tumor‐specific dependencies or reflect perturbation of broadly conserved stress‐response networks. Many of the pathways most commonly affected—particularly those related to redox regulation, apoptosis, and DNA repair—are fundamental to cellular survival in both malignant and non‐malignant contexts. Consequently, therapeutic relevance depends not merely on pathway modulation, but on whether such perturbations exploit context‐specific vulnerabilities within tumor systems. The inherent signaling plasticity of cancer cells (Balk and Goodrich 2024), together with inter‐ and intratumoral heterogeneity, may attenuate the impact of isolated target engagement, especially in the absence of integrated biological validation. Furthermore, the limited consideration of microenvironmental variables (Wang et al. 2025)—such as hypoxia, metabolic adaptation, and stromal interactions—within the reviewed studies constrains extrapolation to complex tumor behavior in vivo. These factors underscore the need to interpret proposed targets within a broader framework of tumor dependency, adaptive capacity, and contextual biological complexity.

This organizational framework provides the foundation for the pathway‐specific analyses that follow. The subsequent sections critically examine each major biological process identified—redox regulation, apoptosis and cell‐cycle control, DNA replication and repair, angiogenesis, and drug resistance—distinguishing between relatively well‐supported mechanistic hypotheses and interactions that likely reflect generalized cellular stress or secondary effects.

### 3.5 Redox Mechanisms and Oxidative Stress: Molecular Targets and Modulation by Metal Complexes

Reactive oxygen species are typically more abundant in cancer cells than in normal tissues due to increased metabolic activity and oncogenic signaling. While moderate ROS levels can promote tumor growth and survival, excessive accumulation leads to oxidative stress and cell death (Ogidi et al. 2025). To maintain redox homeostasis, tumor cells depend on antioxidant enzymes such as thioredoxin reductase (TrxR), glutathione reductase (GR), and catalase (Kim et al. 2019), making redox regulation a widely explored therapeutic vulnerability.

Among the studies included in this review, TrxR is the most frequently investigated redox‐related target. This predominance is strongly supported by chemical considerations, as the selenocysteine residue in its active site is highly susceptible to coordination by soft Lewis acidic metal centers, including Au, Ru, and Pt (Bindoli et al. 2009; Gandin and Fernandes 2015; Salmain et al. 2023). Such interactions, often irreversible, are less accessible to conventional organic compounds and provide a plausible basis for the recurrent observation of TrxR inhibition by metal complexes.

Nevertheless, the biological interpretation of TrxR inhibition remains nontrivial. In many cases, enzyme inhibition demonstrated in vitro is accompanied by increased ROS levels and reduced cell viability, but evidence that TrxR constitutes the primary driver of cytotoxicity is frequently indirect. This complexity is illustrated by the study of Mármol et al. (2019), who reported that alkyl gold complexes derived from 3‐hydroxyflavones inhibited TrxR, GR, and COX‐1/2 activities, inducing apoptosis and oxidative stress in MCF‐7 and HepG2 cells. The observation of compensatory increases in GR activity following TrxR inhibition, alongside multitarget enzyme inhibition by other complexes, suggests adaptive redox responses rather than selective engagement of a single dominant target.

Comparable interpretative limitations apply to catalase, a key enzyme in hydrogen peroxide detoxification (Galasso et al. 2021; Rasheed 2024). Studies by Oveisi Keikha et al. (2023) and Shahraki, Saeidifar et al. (2019) showed that gold and zinc complexes, respectively, reduced catalase activity and correlated with antiproliferative effects. While these findings confirm the susceptibility of antioxidant enzymes to metal‐based compounds, they do not establish catalase inhibition as an independent or primary antitumor mechanism.

Taken together, the reviewed literature associates metal complexes with perturbation of intracellular redox balance and impairment of antioxidant defenses. Within this framework, TrxR appears as the most chemically substantiated target, whereas changes in GR and catalase activity may reflect both direct enzyme interactions and downstream cellular responses to oxidative stress. Overall, these observations highlight the importance of redox pathways in the antitumor activity of metal complexes and reinforce their relevance as mechanistic nodes linking metal coordination chemistry to cancer cell vulnerability.

### 3.6 Regulation of Apoptosis, Cell Cycle and Growth Signaling Pathways

According to Table 2, several metal complexes reported in the reviewed studies have been associated with perturbations in tumor cell survival pathways, particularly those related to apoptosis, cell cycle regulation, and growth signaling. Apoptosis is frequently dysregulated in cancer, enabling malignant cells to evade elimination (Chaudhry et al. 2022). Multiple studies report that metal complexes induce apoptotic features in vitro, including increased oxidative stress, activation of pro‐apoptotic proteins such as Bax, mitochondrial cytochrome c release, caspase activation, and altered expression of BCL‐2 family members (Abdolmaleki et al. 2023). In parallel, computational analyses have suggested potential interactions with antiapoptotic proteins such as MCL‐1, BCL‐2, and BCL‐XL, which may disrupt their binding to BH3 peptides (Lu et al. 2019, 2020), although direct experimental validation remains limited.

For many of these proposed apoptosis‐related targets, however, the available evidence is largely inferential. Docking analyses frequently indicate favorable binding within functional domains, but do not demonstrate intracellular target engagement or establish a causal relationship between protein inhibition and cytotoxicity. This limitation is illustrated by the study of Sarma et al. (2021), in which copper‐based coordination polymers induced apoptosis in tumor cell lines and were subsequently docked against multiple BCL‐2 family proteins. While correlations between predicted binding affinities and cytotoxic potency were observed, the absence of biochemical or cellular target validation precludes definitive attribution of the apoptotic effects to specific antiapoptotic proteins.

Beyond apoptosis regulators, enzymes involved in lipid signaling and stress responses have also been explored. Lipoxygenase, which participates in proliferation and apoptotic regulation (Vishnupriya et al. 2021; Luo et al. 2022), was investigated by Masuri et al. (2023), who reported inhibition of soybean lipoxygenase by a copper complex using enzymatic assays and docking, alongside antiproliferative effects in cancer cells. Although this provides more direct enzymatic evidence, the use of a non‐human enzyme model and limited pathway‐level validation constrain conclusions regarding its relevance as a primary anticancer target.

Cell cycle regulation constitutes another recurrent theme, particularly involving cyclin‐dependent kinases (CDKs), which are frequently dysregulated in cancer and represent validated therapeutic targets (Zhang et al. 2021). Several studies reported inhibition of CDK activity by metal complexes, supported by enzymatic assays, docking, and molecular dynamics simulations (Pravin et al. 2015; Aranda et al. 2020). Some complexes exhibited cytotoxicity comparable to or exceeding that of cisplatin, suggesting functional relevance, although questions regarding intracellular accessibility and mechanistic primacy remain.

Growth signaling pathways and tumor suppressor mechanisms were also addressed. Zinc‐based complexes capable of restoring the functional conformation of mutant p53 through metallochaperone‐like activity represent one of the more mechanistically grounded examples within this category, supported by functional assays demonstrating restored DNA binding and downstream effects (Yu et al. 2017). In contrast, studies targeting membrane and nuclear receptors such as EGFR, HER2, and ERα primarily relied on hybrid designs incorporating known pharmacophores, with computational and cellular data suggesting retained or enhanced receptor inhibition (Konakanchi et al. 2021; Zengin Kurt et al. 2024; Scalcon et al. 2023), but limited direct evidence of receptor engagement.

Proteasome inhibition, particularly targeting the 20S core particle, represents a comparatively well‐established anticancer strategy. The study by Balsa et al. (2021) provides one of the stronger examples within this pathway, combining docking, molecular dynamics, and functional proteasome inhibition assays in cancer cells, although issues of selectivity and off‐target reactivity remain relevant.

The targets grouped within apoptosis, cell cycle, and growth signaling pathways correspond to biologically coherent and clinically relevant processes. The studies reviewed here associate metal complexes with interference with key regulators of tumor cell survival, including apoptotic proteins, cyclin‐dependent kinases, growth factor receptors, and the proteasome. These observations support the view that coordination compounds can interfere with central signaling networks that control proliferation and programmed cell death, highlighting these pathways as important mechanistic contexts for the anticancer activity of metal‐based agents.

### 3.7 Interference With DNA Replication and Repair: Mechanisms and Molecular Targets

Interference with DNA replication and repair is among the most frequently reported biological effects associated with cytotoxic metal complexes. This category includes both direct interactions with DNA and reported inhibition of proteins responsible for maintaining genomic integrity. Although DNA damage often correlates with antiproliferative activity, such effects may arise from multiple mechanisms, including indirect consequences of metal‐induced stress, rather than selective engagement of defined molecular targets (Palermo et al. 2015; Dasmahapatra et al. 2024).

Within this group, the literature predominantly focuses on enzymes that directly regulate DNA topology, particularly topoisomerases. These enzymes play essential roles in resolving topological stress during DNA replication and transcription and are long‐standing targets in anticancer therapy (McKie et al. 2021; Mastrangelo et al. 2022). Several metal complexes have been reported to interfere with topoisomerase activity by stabilizing cleavage complexes and preventing DNA religation, leading to DNA strand breaks and cell cycle arrest or apoptosis (Gaikwad et al. 2022). However, evidence for topoisomerases as primary intracellular targets often relies on in vitro enzymatic inhibition, which does not necessarily demonstrate selective or biologically dominant target engagement.

This limitation is illustrated by studies such as those of Akinyemi et al. (2023), who observed inhibition of topoisomerase Iβ by palladium complexes in gel‐based assays, and Chatterjee et al. (2023), who reported a copper(II)–quinalizarin complex as a dual inhibitor of topoisomerases I and II supported by docking analyses. While consistent with topoisomerase interference, these findings do not conclusively establish causal links between enzyme inhibition and cytotoxicity at the cellular level.

Beyond topoisomerases, fewer studies have addressed other components of DNA synthesis and repair. Ribonucleotide reductase (RNR), a key enzyme in deoxyribonucleotide biosynthesis (Marcus et al. 2022), was investigated as a target of copper morpholine–thiosemicarbazone complexes, with docking and tyrosyl radical reduction assays suggesting moderate inhibition, potentially related to iron chelation (Ohui et al. 2018). Similarly, poly(ADP‐ribose) polymerases, particularly PARP1, were explored through metal complexes incorporating PARP‐inhibitory ligands. Ruthenium(II) and iridium(III) complexes inhibited PARP‐1 activity in vitro and induced cell cycle arrest, apoptosis, and DNA damage markers in cancer cells (Pavlović et al. 2020; Yang et al. 2023). Nonetheless, whether PARP1 inhibition represents a dominant mechanism or part of a broader cellular response remains unclear.

Proteins involved in DNA replication and repair constitute mechanistically appealing targets for metal‐based anticancer agents. The studies reviewed describe interference with key regulators of genomic integrity, including topoisomerases, ribonucleotide reductase, and PARP enzymes, whose inhibition or functional interference is frequently associated with DNA damage responses, cell cycle arrest, and apoptosis in cancer cells. These observations support the relevance of DNA metabolism as an important mechanistic context for the biological activity of metal complexes and reinforce the central role of genome maintenance pathways in mediating their antiproliferative effects.

### 3.8 Inhibition of Tumor Angiogenesis: Strategic Targets

Angiogenesis is a neovascularization process that is essential for tumor growth and dissemination, as it enables the supply of oxygen and nutrients to cancer cells (Ozel et al. 2022). This process is regulated by multiple molecular mediators, among which Vascular Endothelial Growth Factor Receptor 2 (VEGFR‐2) is widely regarded as a central regulator, alongside proteins such as angiogenin and Girdin, which contribute to endothelial proliferation, migration, and cytoskeletal dynamics (Shah et al. 2021; Mao et al. 2024; Hayashi et al. 2023). Owing to their roles in angiogenic signaling, these proteins have been explored as potential targets for metal complexes; however, the extent to which their reported inhibition or alteration translates into consistent and selective antiangiogenic responses remains unevenly supported across studies.

In this context, Abdalrazaq et al. (2024) proposed VEGFR‐2 as a potential target for zinc and cadmium dithiocarbamate complexes based on molecular docking analyses. The complexes exhibited interaction energies comparable to or exceeding those of the reference inhibitor sorafenib and formed hydrophilic interactions with residues GLU885 and ASP1046, which are critical for VEGFR‐2 inhibition (McTigue et al. 2012). While these results suggest structural compatibility with the VEGFR‐2 binding site, the evidence remains purely theoretical, underscoring the need for experimental validation to determine whether such interactions occur in cellular systems and whether they result in functionally relevant inhibition of angiogenesis.

A more direct protein‐level interaction was reported by Marzo et al. (2022), who demonstrated that oxaliplatin inhibits angiogenin‐mediated cell proliferation and migration in PC‐3 prostate cancer cells. Reactivity between oxaliplatin and angiogenin was confirmed by electrospray ionization mass spectrometry (ESI‐MS), and the protein–ligand complex structure was resolved by X‐ray crystallography. This finding is notable, as it reveals an additional molecular interaction for an FDA‐approved drug (Ibrahim et al. 2004). Nevertheless, given the well‐documented adverse effects associated with oxaliplatin (O'Dowd et al. 2023), these results primarily highlight angiogenin as a potentially druggable target rather than establishing a therapeutically favorable anti‐angiogenic strategy per se, motivating the exploration of alternative metal complexes with improved selectivity and reduced toxicity.

Girdin has been investigated even more sparsely as a metallodrug target. The only study identified in this review was conducted by Ruan et al. (2024), who proposed an iridium(III) complex (1a) as an inhibitor of this protein. Target engagement was initially suggested by thermal proteome profiling (TPP) and further supported by cellular thermal shift assays (CETSA), with docking studies indicating binding within a hydrophobic pocket of Girdin. The complex displayed cytotoxic activity exceeding that of cisplatin in breast and lung cancer cell lines and organoid models and was associated with inhibition of the EGFR/AKT/mTOR/STAT3 signaling pathway. While these findings position Girdin as an intriguing and underexplored target, the limited number of studies and the complexity of downstream signaling effects warrant caution in attributing the observed cytotoxicity solely to Girdin inhibition.

Angiogenesis‐related proteins represent biologically relevant molecular targets or interaction partners for metal‐based compounds, given their central roles in tumor vascularization and endothelial cell regulation. The studies reviewed describe reported interactions with proteins such as angiogenin, VEGFR‐2, and Girdin, which have been associated with effects on endothelial proliferation, migration, and angiogenic signaling pathways. Among these, angiogenin currently represents the most structurally and experimentally characterized case, whereas investigations involving VEGFR‐2 and Girdin remain limited. Consequently, despite their mechanistic relevance in tumor vascularization, these targets should still be regarded as exploratory and context‐dependent, highlighting angiogenesis as a promising but still underdeveloped area for mechanistic investigation of metal‐based anticancer agents.

### 3.9 Overcoming Drug Resistance: Approaches Based on Metal Complexes

Drug resistance remains a major challenge in cancer therapy and is frequently associated with cellular mechanisms that reduce intracellular drug accumulation or promote detoxification (Vijayakumar et al. 2024). Among the most investigated targets in this context are P‐glycoprotein (P‐gp) and glutathione S‐transferase π (GSTπ). P‐gp functions as an ATP‐dependent efflux pump that lowers intracellular drug concentrations (Guo et al. 2024), whereas GSTπ participates in the conjugation of chemotherapeutic agents with glutathione, contributing to cellular detoxification (Ściskalska and Milnerowicz 2020). Their overexpression in tumor cells is commonly associated with reduced therapeutic response, supporting their relevance as resistance‐related targets.

Côrte‐Real et al. (2019) reported a ruthenium complex with cytotoxic activity in MCF7 and MDA‐MB‐231 cells and selective inhibition of P‐gp. Inhibition was evaluated through MTT assays in cell lines genetically modified to overexpress ABC transporters and by flow cytometry measuring intracellular accumulation of fluorescent substrates. Molecular docking analyses indicated favorable binding energies between the complex and P‐gp. Together, these findings support functional interference with P‐gp activity, although the extent to which this interaction alone accounts for the observed cytotoxicity cannot be definitively established.

Inhibition of GSTπ by metal complexes was examined by Lin et al. (2015), who investigated the binding of organometallic ruthenium compounds to the enzyme. Using electrospray ionization mass spectrometry (ESI‐MS), the authors quantified binding stoichiometry and identified Cys15 and Cys48, located in the G site, as the principal coordination sites associated with enzymatic inhibition. Binding to residues at the dimer interface, such as Met92 and Cys102, contributed minimally to inhibition. The presence of glutathione reduced binding at interface residues but did not significantly affect coordination at Cys15 and Cys48. These results provide direct biochemical evidence of site‐specific interaction consistent with GSTπ inhibition.

Targeting resistance‐associated proteins such as P‐gp and GSTπ represents a therapeutically relevant strategy aimed at restoring or enhancing cellular sensitivity to treatment rather than directly interfering with core proliferative or tumorigenic signaling pathways. Unlike proteins that govern angiogenesis, cell cycle progression, or DNA replication, these targets are primarily associated with adaptive mechanisms that can emerge during therapeutic exposure. Although functional and biochemical studies indicate that certain metal complexes can bind to or inhibit these proteins, the number of investigations remains limited. Consequently, P‐gp and GSTπ should currently be regarded as exploratory and context‐dependent targets, highlighting drug resistance pathways as a promising but still developing area for the mechanistic study of metal‐based anticancer agents.

### 3.10 Pharmacological and Target‐Selectivity Considerations

Across the different target classes discussed above, an additional overarching consideration concerns the intrinsic reactivity of many metal complexes toward nucleophilic biomolecules. The coordination properties that enable strong interactions with specific proteins—particularly through cysteine and selenocysteine residues—also increase the likelihood of broader protein engagement within the cellular environment. Given the abundance of thiol‐containing molecules, including glutathione and cysteine‐rich proteins, selective target engagement may compete with nonspecific binding and intracellular sequestration processes, which may also contribute to reduced selectivity and potential systemic toxicity (Jiang et al. 2023; Rodrigues et al. 2024).

In this context, the strong coordination chemistry of many metal complexes frequently results in covalent or quasi‐irreversible interactions with nucleophilic residues. While covalent or quasi‐irreversible coordination can enhance potency and prolong target interaction—a well‐established strategy to maximize drug‐target residence time—it can also reduce control over the duration of target engagement and contribute to off‐target effects. As discussed in the literature, excessively long residence times can be associated with adverse events, thereby narrowing the therapeutic window (Knockenhauer and Copeland 2023). Consequently, enzymatic inhibition observed under simplified experimental conditions does not necessarily translate to selective intracellular engagement, particularly in complex biological systems where metal speciation and ligand exchange dynamically influence bioavailability.

In addition to these pharmacological considerations, several methodological aspects of the reviewed studies also influence how reported targets should be interpreted. Much of the available evidence derives from in vitro enzymatic assays, docking analyses, or simplified biochemical systems that do not fully capture intracellular complexity. In particular, metal complexes may generate assay artifacts through redox activity (Proj et al. 2022) or nonspecific interactions with biomolecules present in the assay environment (Forero et al. 2023). Moreover, many targets discussed across the previous sections—especially those related to angiogenesis and drug resistance—are supported by a limited number of studies, often combining indirect biochemical observations with computational predictions. Under these conditions, distinguishing direct target engagement from broader cellular responses, such as oxidative stress, DNA damage, or metabolic disruption, becomes difficult. Consequently, many of the proteins proposed as targets should be regarded as preliminary or context‐dependent interaction points rather than definitively established molecular drivers of the observed anticancer effects.

These considerations are especially relevant when interpreting mechanistic claims across the reviewed literature. Many reported targets are supported by biochemical or computational evidence but remain insufficiently validated within physiologically relevant contexts. As a result, the distinction between primary drivers of cytotoxicity and downstream consequences of broader cellular stress responses remains challenging. Recognizing these pharmacological constraints does not diminish the therapeutic promise of metal complexes; rather, it highlights the need to interpret target identification within the broader framework of cellular chemistry, biological competition, and systemic exposure, which ultimately govern translational success.

### 3.11 Limitations of Study

This systematic review has important methodological and conceptual limitations. First, study selection was conducted by a single reviewer, which deviates from the preferred practice of dual independent screening. Second, due to the substantial heterogeneity in experimental design and target validation strategies among the included studies, a formal Risk of Bias assessment was not feasible; instead, a Methodological Quality Assessment was applied.

Additionally, the available evidence is predominantly based on in vitro models and indirect indicators of target engagement, limiting the strength of causal inferences and translational interpretation. The possibility of publication bias toward positive findings cannot be excluded and should be considered when interpreting the conclusions of this review.

## 4 Conclusions

This systematic review mapped the protein targets most frequently associated with the antitumor activity of metal complexes and critically examined the nature of the supporting evidence. The literature is predominantly centered on pathways related to redox homeostasis, apoptosis, DNA replication, and cell cycle regulation, with additional exploration of angiogenesis and resistance‐associated proteins. Collectively, these targets reflect biologically relevant processes in tumor progression and treatment response.

However, across these categories the strength of mechanistic inference varies considerably. Most reported targets are supported primarily by in vitro assays and computational analyses, without validation in animal models within the body of literature examined here. Consequently, several proposed protein interactions should be regarded as biologically coherent yet still preliminary with respect to definitive target validation and translational applicability.

Overall, the available evidence suggests that metal complexes can perturb multiple survival‐related cellular networks, but distinguishing therapeutically actionable, tumor‐relevant targets from broader stress‐associated effects remains a central challenge for the field. Addressing this limitation will require the integration of more robust experimental strategies capable of validating intracellular target engagement and clarifying the causal relationship between protein interaction and antitumor activity.

## Author Contributions

**Allysson L. dos S. Ferreira:** conceptualization, methodology and analysis, writing – original draft. **Bruna B. Dantas:** writing – review and editing. **Jailton De Souza‐Ferrari:** writing – review and editing. **Edilson B. Alencar Filho:** conceptualization, supervision, writing – review and editing. All authors reviewed and approved the final version of the manuscript.

## Conflicts of Interest

The authors declare no conflicts of interest.

## Supporting information


**Table S1:** Search strategy and electronic information sources. **Table S2:** Molecular targets associated with the antitumor activity of metal complexes identified in the literature. **Table S3:** Study‐level methodological quality assessment of included studies evaluating protein targets of anticancer metal complexes.

Supporting File

## Data Availability

The data that support the findings of this study are available from the corresponding author upon reasonable request.
