# Supplementary material for: Potential Biological Targets of Anticancer Metal‐Based Drug Candidates: A Systematic Review
Source: Drug Dev Res. 2026 May 18;87:e70315. doi: 10.1002/ddr.70315 (PMC13181534; doi:10.1002/ddr.70315)
Supplement: Supplementary file 1 — Table S1: Search strategy and electronic information sources. Table S2: Molecular targets associated with the antitumor activity of metal complexes identified in the literature. Table S3: Study‐level methodological quality assessment of included studies evaluating protein targets of anticancer metal complexes. [file DDR-87-e70315-s001.docx]

**Potential Biological Targets of Anticancer Metal-Based Drug Candidates: A Systematic Review**

**Supplementary material**

**Table S.1 - Search strategy and electronic information sources.**

| **Information source** | **Search strategy** | **Manuscripts** |
| --- | --- | --- |
| ScienceDirect | ("coordination complexes" OR metallodrugs) AND ("anticancer activity") AND (docking OR "molecular dynamics" OR "mechanism of action") | 559 |
| Scopus | ( TITLE-ABS-KEY ( "coordination complexes" ) OR TITLE-ABS-KEY ( metallodrugs ) ) AND ( TITLE-ABS-KEY ( "anticancer activity" ) OR TITLE-ABS-KEY ( "antineoplastic agents" ) ) AND ( TITLE-ABS-KEY ( docking ) OR TITLE-ABS-KEY ( "molecular dynamics" ) OR TITLE-ABS-KEY ( "mechanism of action" ) OR TITLE-ABS-KEY ( "target identification" ) OR TITLE-ABS-KEY ( "binding affinity" ) ) AND PUBYEAR > 2014 AND PUBYEAR < 2026 AND PUBYEAR > 2014 AND PUBYEAR < 2026 AND ( LIMIT-TO ( DOCTYPE , "ar" ) ) AND ( LIMIT-TO ( SUBJAREA , "BIOC" ) OR LIMIT-TO ( SUBJAREA , "PHAR" ) OR LIMIT-TO ( SUBJAREA , "CHEM" ) OR LIMIT-TO ( SUBJAREA , "MULT" ) ) AND ( LIMIT-TO ( EXACTKEYWORD , "Human Cell" ) OR EXCLUDE ( EXACTKEYWORD , "DNA Binding" ) OR EXCLUDE ( EXACTKEYWORD , "DNA Damage" ) OR EXCLUDE ( EXACTKEYWORD , "Down Regulation" ) OR EXCLUDE ( EXACTKEYWORD , "Protein Expression" ) OR EXCLUDE ( EXACTKEYWORD , "Drug DNA Interaction" ) ) | 208 |
| Web of Scienc | TS=("coordination complexes" OR metallodrugs)  AND TS=("anticancer activity" OR "antineoplastic agents")  AND TS=(docking OR "molecular dynamics" OR "mechanism of action" OR "target identification" OR "binding affinity")  AND PY=(2015-2025) | 68 |
| Wiley | ("coordination complexes" OR metallodrugs) AND ("anticancer activity" OR "antineoplastic agents") AND (docking OR "molecular dynamics" OR "mechanism of action" OR "target identification" OR "binding affinity") NOT (DNA OR "DNA binding" OR "gene expression" OR "DNA damage" OR "down regulation" OR "drug-DNA interaction") | 38 |

Source: By the author, 2026.

**Tabela S.2- Molecular targets associated with the antitumor activity of metal complexes identified in the literature.**

| **Molecular target** | **Number of studies** | **Metal Center (s)** | **Proposed mechanism of action related to the target** | **Level of Evidence** | **References** |
| --- | --- | --- | --- | --- | --- |
| Thioredoxin reductase (TrxR) | 9 | Au, Ag, Cu, Ru | Target inhibition  Cellular redox imbalance  Increased ROS levels  Induction of apoptosis | Strong | (Sánchez-de-Diego et al., 2017; Dominelli et al., 2020; Zare et al., 2025; Tolbatov et al., 2021; Mármol et al., 2019; Scalcon et al., 2023; Tabrizi; Chiniforoshan, 2016; Yu et al., 2024; Tolbatov et al., 2024) |
| Topoisomerase I/II | 6 | Pd, Pt, Cu, Ag, Zn, V | Targets inhibition  Inhibits DNA repair  Cell cycle arrest  Induction of apoptosis | Strong | (Akinyemi et a., 2023; Chatterjee et al., 2023; Reig et al., 2018; Mariconda et al., 2022; Gaur et al., 2017; León et al., 2015) |
| B-cell lymphoma 2 family (BCL-2) | 6 | Ni, Cu, Ag, Mn, Co, Zn, Ru | Targets inhibition  Activation of the intrinsic pathway  Induction of apoptosis | Moderate | (Sharma et al., 2021; Varna et al., 2023; Baishya et al., 2022; Nashre-ul-Islam et al., 2019; Sarma et al., 2021; Rana et al., 2021) |
| Bovine Liver Catalase (BCL) | 5 | Au, Pd, Pt, Zn | Target inhibition  Accumulation of ROS  Increased oxidative stress  Induction of apoptosis | Strong | (Keikha et al., 2023; Shahraki et al., 2019a; Keikha et al., 2024; Shahraki et al., 2019b; Shahraki et al., 2025) |
| Epidermal Growth Factor Receptor (EGFR) | 5 | Co,Ni, Ru, Cu, Zn e Pd | Target inhibition  Blockade of proliferative signaling  Cell cycle arrest  Induction of apoptosis | Strong | (Yu et al., 2024; Gogoi et al., 2019; Kurt et al., 2024; Ghorbanpour et al., 2022; Konakanchi et al., 2021) |
| Cyclin-dependent kinases (CDK1, CDK2) | 3 | Cu, Pt e Zn | Targets inhibition  Cell cycle dysregulation  Checkpoint arrest (G1/S or G2/M)  Induction of apoptosis | Moderate | (Ghorbanpour et al., 2022; Aranda et al., 2020; Pravin et al., 2015) |
| 20S proteasome | 3 | Cu, Ru | Target inhibition  Accumulation of damaged proteins  Induction of apoptosis | Strong | (Balsa et al., 2021; El Yaagoubi et al., 2024; Cuccioloni et al., 2019) |
| Cathepsin B | 2 | Fe, Pd | Target inhibition  Lysosomal dysfunction  Activation of apoptotic pathways | Moderate | (Akinyemi et al., 2023; Garcia et al., 2022) |
| Glutathione reductase (GR) | 2 | Au, Cu | Target inhibition  Cellular redox imbalance  Increased ROS levels  Induction of apoptosis | Moderate | (Mármol et al., 2019; Scalcon et al., 2023) |
| Pyruvate kinase M2 (PKM2) | 2 | Fe | Target modulation  Alteration of glycolytic metabolism  Affects proliferation and immune response | Moderate | (Jadhav et al., 2022; Gupta et al., 2022) |
| Poly(adenosine diphosphate-ribose) polymerase (PARP) | 2 | Ru, Ir | Target inhibition  Inhibits DNA repair  Accumulation of DNA strand breaks  Cell cycle arrest  Induction of apoptosis | Moderate | (Pavlović et al., 2020; Yang et al., 2023) |
| Lipoxygenase (LOX) | 1 | Cu, Ag, Sb | Allosteric inhibition  Suppression of tumor proliferation  Induction of apoptosis | Preliminary | (Masuri et al., 2023) |
| Vascular endothelial growth factor receptor 2 (VEGFR-2) | 1 | Zn, Cd | Target inhibition  Inhibition of angiogenesis  Reduction of cell migration  Suppression of tumor growth | Preliminary | (Abdalrazaq et al., 2024) |
| ADP-sugar pyrophosphatase (NUDT5) | 1 | V, Sn | Target inhibition with differential expression associated with breast cancer | Preliminary | (Thakur et al., 2024) |
| 7β-hydroxysteroid dehydrogenase (7β-HSD) | 1 | V, Sn | Target inhibition with differential expression associated with breast cancer | Preliminary | (Thakur et al., 2024) |
| p53 | 1 | Zn | Zinc delivery to mutant p53  Restoration of tumor suppressor function | Preliminary | (Yu et al., 2017) |
| Tubulin | 1 | Pt | Target inhibition  Cytoskeletal disorganization  Cell cycle arrest  Induction of apoptosis | Preliminary | (Kostrhunova et al., 2019) |
| BRAF kinase | 1 | Ni | Target inhibition with differential expression associated with cancer progression | Preliminary | (Gogoi et al., 2019) |
| Chemokine receptor 9 (CCR9) | 1 | Ni | Target inhibition with differential expression associated with cancer progression | Preliminary | (Gogoi et al., 2019) |
| Phosphatidylinositol 3-kinase (PI3K) | 1 | Ni | Target inhibition with differential expression associated with cancer progression | Preliminary | (Gogoi et al., 2019) |
| Aldehyde dehydrogenase (ALDH) | 1 | Ru | Target inhibition with differential expression associated with cancer progression | Preliminary | (Caruso et al., 2022) |
| Angiogenin | 1 | Pt | Target inhibition  Inhibition of angiogenesis  Reduction of tumor proliferation | Preliminary | (Marzo et al., 2022) |
| Cyclooxygenases (COX-1/2) | 1 | Au | Target inhibition  Suppression of tumor proliferation | Preliminary | (Mármol et al., 2019) |
| Alkaline phosphatase (ALP) | 1 | Cu, Pt | Inhibition of histone phosphorylation  Modulation of chromatin remodeling  Regulation of DNA accessibility | Preliminary | (Aranda et al., 2020) |
| Human glutathione S-transferase pi (GSTπ) | 1 | Ru | Function not explored in this study. For context on the target's activity in cancer, see (Ściskalska & Milnerowicz, 2020) | Preliminary | (Lin et al., 2015) |
| P-glycoprotein (P-gp) | 1 | Ru | Inhibition of the efflux pump  Reduction of drug resistance | Preliminary | (Côrte-Real et al., 2019) |
| Estrogen receptor alpha (ERα) | 1 | Au, Cu | Inhibition of receptor activity  Alteration of hormonal signaling  Influence on cell proliferation | Preliminary | (Scalcon et al., 2023) |
| Girdin | 1 | Ir | Target inhibition  Inhibition of proliferation and metastasis | Preliminary | (Ruan et al., 2024) |
| Cathepsin D | 1 | Cu | Target inhibition related to prostate cancer  Cell cycle arrest and key signaling pathways suppressed | Preliminary | (Qu et al., 2022) |
| Protein disulfide isomerase (PDI) | 1 | Cu | Target inhibition with differential expression associated with cancer | Preliminary | (Miglioli et al., 2024) |
| Kinases (SGK-1, PKA, CaMK-1, MSK1, and GSK3β) | 1 | Cu | Inhibition of multiple kinases regulating tumor progression | Preliminary | (Wittmann et al., 2022) |
| Aldo-keto reductases (AKR1C1–1C3) | 1 | Ru | Target inhibition in hormone-dependent cancers (e.g., breast, prostate, and endometrial) | Preliminary | (Traven et al., 2014) |
| Histone deacetylase (HDAC) | 1 | Fe | HDAC inhibition with reactivation of ERα and sensitization to hormonal treatment | Preliminary | (Tang et al., 2018) |
| Calcium ATPase (Ca ATPase) | 1 | V | Inhibition of Ca²⁺ ATPase and antiproliferative effect | Preliminary | (De Souza Coelho et al., 2022) |
| AFF4–CCNT1 | 1 | Ir | Inhibition of the AFF4–CCNT1 pathway associated with various cancers | Preliminary | (Song et al., 2024) |
| Ribonucleotide reductase (RNR) | 1 | Cu | RNR inhibition associated with DNA synthesis/repair damage | Preliminary | (Ohui et al., 2018) |
| HMG-CoA reductase | 1 | Ru | Target inhibition  Disruption of cholesterol synthesis | Preliminary | (Cuccioloni et al., 2019) |
| Human epidermal growth factor receptor 2 (HER2) | 1 | Co, Ni, Cu, Zn e Pd | Target inhibition with differential expression associated with cancer | Preliminary | (Konakanchi et al., 2021) |
| Citocromo c (cyt c) | 1 | Pt, Pd | Function not explored in this study. For context on the target's activity in cancer, see (Alshehri, 2024) | Preliminary | (Savić et al., 2018) |
| Ribonuclease A (RNase A) | 1 | Pt, Pd | Function not explored in this study. For context on the target's activity in cancer, see (Abbasi et al., 2023) | Preliminary | (Savić et al., 2018) |

The 'Level of Evidence' was classified as follows:

Strong: Supported by three or more studies, with at least two providing direct experimental evidence of target engagement (e.g., enzymatic inhibition assay, biophysical characterization).

Moderate: Supported by two studies, or by multiple studies relying primarily on computational or indirect cellular evidence.

Preliminary: Supported by a single study.

Source: By the author, 2026.

**Table S.3 - Study-level methodological quality assessment of included studies evaluating protein targets of anticancer metal complexes.**

| Study (Authors,year) | Direct Experimental Evidence | Supporting Computational Evidence | Correlation with Biological Activity |
| --- | --- | --- | --- |
| (Keikha et al., 2023) | Y | Y | Y |
| (Thakur et al., 2024) | N | Y | N |
| (Garcia et al., 2022) | Y | Y | Y |
| (Sánchez-de-Diego et al., 2017) | Y | N | Y |
| (Yu et al., 2017) | Y | N | Y |
| (Dominelli et al., 2020) | Y | N | Y |
| (Kostrhunova et al., 2019) | Y | N | Y |
| (Gogoi et al., 2019) | N | Y | N |
| (Masuri et al., 2023) | Y | Y | Y |
| (Kurt et al., 2024) | Y | Y | Y |
| (Caruso et al., 2022) | Y | Y | Y |
| (Abdalrazaq et al., 2024) | N | Y | N |
| (Reig et al., 2018) | Y | Y | Y |
| (Keikha et al., 2024) | Y | Y | Y |
| (Shahraki et al., 2019a) | Y | Y | Y |
| (Shahraki et al., 2025) | Y | Y | Y |
| (Sharma et al., 2021) | N | Y | Y |
| (Shahraki et al., 2019b) | Y | Y | Y |
| (Zare et al., 2025) | N | Y | Y |
| (Tabrizi; Chiniforoshan, 2016) | Y | N | Y |
| (Aranda et al., 2020) | Y | N | Y |
| (Lin et al., 2015) | Y | N | Y |
| (Côrte-Real et al., 2019) | Y | Y | Y |
| (Akinyemi et al., 2023) | Y | N | Y |
| (Jadhav et al., 2022) | Y | Y | Y |
| (Balsa et al., 2021) | Y | Y | Y |
| (Scalcon et al., 2023) | Y | Y | Y |
| (Ruan et al., 2024) | Y | Y | Y |
| (Qu et al., 2022) | Y | Y | Y |
| (Chatterjee et al., 2023) | Y | Y | Y |
| (Ghorbanpour et al., 2022) | N | Y | N |
| (Miglioli et al., 2024) | Y | N | Y |
| (Wittmann et al., 2022) | Y | Y | Y |
| (El Yaagoubi et al., 2024) | Y | N | Y |
| (Varna et al., 2023) | N | Y | Y |
| (Mariconda et al., 2022) | Y | Y | Y |
| (Ohui et al., 2018) | N | Y | N |
| (Cuccioloni et al., 2019) | Y | N | Y |
| (Pavlović et al., 2020) | Y | N | Y |
| (Yu et al., 2024) | Y | N | Y |
| (Traven et al., 2014) | Y | N | Y |
| (Tolbatov et al., 2024) | N | Y | N |
| (Baishya et al., 2022) | N | Y | Y |
| (Tang et al., 2018) | Y | Y | Y |
| (De Souza Coelho et al., 2022) | N | N | Y |
| (Song et al., 2024) | Y | Y | Y |
| (Yang et al., 2023) | Y | Y | Y |
| (Gupta et al., 2022) | Y | Y | Y |
| (Nashre-ul-Islam et al., 2019) | N | Y | Y |
| (Sarma et al., 2021) | N | Y | Y |
| (Rana et al., 2021) | N | Y | Y |
| (Marzo et al., 2022) | Y | N | Y |
| (Tolbatov et al., 2021) | N | Y | N |
| (Mármol et al., 201) | Y | N | Y |
| (Gaur et al., 2017) | Y | Y | Y |
| (León et al., 2015) | Y | N | Y |
| (Konakanchi et al., 2021) | N | Y | Y |
| (Savić et al., 2018) | Y | N | Y |
| (Pravin et al., 2015) | N | Y | N |

Abbreviations: Y, Yes; N, No.

Source: By the author, 2026.
